# Association of Family History of Type 2 Diabetes with Prostate Cancer: A National Cohort Study

**DOI:** 10.3389/fonc.2016.00194

**Published:** 2016-08-29

**Authors:** Jianguang Ji, Jan Sundquist, Kristina Sundquist

**Affiliations:** ^1^Center for Primary Health Care Research, Lund University, Malmö, Sweden

**Keywords:** prostate cancer, familial risk, incidence, type 2 diabetes

## Abstract

**Background:**

Personal history of type 2 diabetes mellitus (T2DM) is associated with a lower incidence of prostate cancer, but the underlying mechanisms are largely unknown. We hypothesized that genetic factors that are involved in the development of T2DM might protect against prostate cancer.

**Methods:**

We used a few Swedish registers, including the Swedish Multigeneration Register and the Cancer Register, to examine the risk of prostate cancer among men with a family history of T2DM. Standardized incidence ratios were used to calculate the relative risk.

**Results:**

The overall risk of prostate cancer among men with a familial history of T2DM was 0.87 (95% CI: 0.86–0.89) as compared to matched controls. The risk was even lower for those multiple affected relatives with T2DM, and it was 0.86 for those with two affected relatives and 0.67 for those with three and more affected relatives.

**Conclusion:**

Family history of T2DM was associated with a lower incidence of prostate cancer, and the risk was even lower for those with more than one affected relative. Our study strongly suggests that genetic factors or shared familial factors, such as obesity, that contributed to T2DM may protect against prostate cancer.

## Introduction

The incidence of type 2 diabetes mellitus (T2DM) has continuously increased worldwide and in Sweden (1), partly due to the increasing trend of obesity, which is one of the main risk factor of T2DM (2). Personal history of T2DM has consistently reported to be associated with an increased incidence and mortality of various types of cancer (3, 4), with one exception of prostate cancer (5–7). The underlying mechanisms are still largely unknown. Lower detection rate of prostate cancer due to altered health care seeking behaviors in T2DM, such as PSA examination, has been suggested to contribute to the inverse association (5, 8). The US Multiethnic Cohort study found that the frequency of PSA testing is around 44% in diabetics, whereas the frequency is 48 in non-diabetics (8). Other factors, such as low-androgen level in T2DM as well as the protective effects of diabetes medication (9), may also contribute to the lower incidence of prostate cancer. However, it is still unknown whether genetic factors that are involved in the development of T2DM might protect against prostate cancer. Shared genetic component between T2DM and prostate cancer has been investigated in many previous studies by using multiple single-nucleotide polymorphisms. Pierce and Ahsan found that the genetic score of T2DM based on 18 SNPs showed an inverse association with prostate cancer (10). Another study using individual SNPs and aggregations of 36 T2DM susceptibility loci found an association with prostate cancer (11). In the current study, we explored the hypothesis that genetic factors may partly explain the inverse association between T2DM and prostate cancer by examining the incidence of prostate cancer among Swedish men with a family history of T2DM as compared to men without a family history.

## Patients and Methods

This cohort study was approved by the Regional Ethical Review Board of Lund University, Sweden in 2013. This study was carried out by using several nationwide Swedish Registers. The Swedish Cancer Register, which was founded in 1958 by the National Board of Health and Welfare and has almost complete nationwide coverage (12). All physicians in Sweden must report all cases of cancer to the Swedish Cancer Registry according to instructions by the National Board of Health and Welfare based on clinical and pathological reports (13). The majority of cancer cases were notified twice from separate reports, guaranteeing a high accuracy rate at a national level. A 4-digit diagnosis code according to the 7th revision of the International Classification of Diseases (ICD-7) has been used in the Swedish Cancer Register. The Multigeneration Register (14), which was created and maintained by Statistics Sweden, includes all children born in Sweden in 1932 and later (maximally 80 years old at 2012) and their siblings and biological parents. More than 14.4 million individuals (living and deceased) in more than 3.8 million families were included in the Multigeneration Register. The Swedish Hospital Discharge Register, which was founded in 1964 by the National Board of Health and Welfare and has had complete national wide coverage since 1987 (15), and the Swedish Outpatient Register, founded in 2001 with complete coverage (16), were used to identify a cohort of patients with T2DM. Diagnoses of diabetes were reported according to the different versions of ICD codes. ICD-9 code of 250 was used to retrieve patients diagnosed with diabetes in years between 1987 and 1996; ICD-10 code of E11 was used between 1997 and 2012. The quality of the Swedish Hospital Register has been examined extensively (17). As compared to the diagnoses from medical records, the positive predictive values (PPV) are generally 85–95%.

Additional linkages were made to the Swedish National Population and Housing Census (18) to obtain information on individual-level characteristics, such as year of birth, gender, socioeconomic status, and region of residence; to the Cause of Death Register to identify date of death; to the Emigration Registry to identify date of emigration. All linkages were performed using individual national identification numbers, which were replaced with serial numbers in order to preserve anonymity.

### Study Population

The study population was men who were born between 1932 and 1957 and were still alive in 1987 (age ranging between 30 and 55 at the beginning of study). Using the hospital records, we identified all the men who had a family history of T2DM. Five men from the general population without a family history of T2DM were matched according to year of birth, socioeconomic status, and regions of residence. Individuals who were diagnosed with cancer before 1987 were excluded from the current study.

### Outcome Variable

The Swedish Cancer Register recorded both the sites and histological types of cancer. Prostate cancer was defined by the ICD-7 code of 177. Only the first primary prostate cancer was considered in the present study.

### Predictive Variable

Familial history of T2DM was identified if individuals had one or more first degree relatives (parents and siblings) diagnosed with T2DM between 1987 and 2012. T2DM and type 1 diabetes mellitus (T1DM) were first distinguished from the Swedish Hospital Register in 1997 by using ICD-10 codes. To guard against inclusion of T1DM patients diagnosed between 1987 and 1996, we used an age at diagnosis of diabetes over 39 years to define T2DM during 1987 and 1996, as is done in the Swedish Diabetes Registry.

### Individual-Level Variables Adjusted in the Model

Other variables that were associated with prostate cancer included age and period at diagnosis, which was categorized into 5-year groups, socioeconomic status, and regions of residence. We classified each individual’s socioeconomic status into one of six categories: (1) farmer, (2) manual worker, (3) blue collar, (4) professional, (5) private, and (6) other. Geographic region of residence was divided into large cities (those with a population of >200,000, i.e., Stockholm, Gothenburg, and Malmö), Southern Sweden, Northern Sweden, and unknown.

### Statistical Analysis

Person-years at risk (number of persons at risk multiplied by time at risk) were calculated from the start of follow-up on 1 January 1987 until the diagnosis of cancer or death, emigration or the end of follow-up (31 December 2012). Standardized incidence ratios (SIRs) were calculated as the ratio of observed to expected number of cases. SIRs were used to measure the relative risk of prostate cancer in men with a family history of T2DM compared with matched controls. The expected number of cases was calculated for age (5-year groups), follow-up interval (5-year groups), socioeconomic status, and region of residence-specific standard incidence rates derived from individuals lacking an affected family member (19). Ninety-five percent confidence intervals were calculated assuming a Poisson distribution. Data values were accurate to two decimal places.

In our exposure definition, all participants with deceased parents before 1987 would be classified as having no family history of T2DM, which may lead to misclassification of exposure. We, thus, calculated the adjusted SIR in the Table A1 in Appendix based on the percentage of misclassification of the exposure in the control group. All analyses were performed using SAS^®^ version 9.2 (SAS Institute, Cary, NC, USA).

## Results

A total of 198,129 men were retrieved from the databases with a family history of T2DM (Table 1). The median age was 41 at the beginning of follow-up (year 1987). The median follow-up time was 24 years. During the study period, 4.6% of them with a family history of T2DM were diagnosed with prostate cancer, whereas the proportion was 5.3% for those without a family history.

**Table 1:** **Basic characteristics among men with a family history of type 2 diabetes and matched control**.

Basic characteristics	With family history		Matched control	
	Number	%	Number	%
Age				
30–39	82,239	41.5	411,195	41.5
40–49	88,825	44.8	444,125	44.8
≥50	27,065	13.7	135,325	13.7
Median (year)	41		41	
Socioeconomic status				
Farmer	4012	2.0	20,060	2.0
Manual worker	77,703	39.2	388,515	39.2
Blue collar	48,426	24.4	242,130	24.4
Professional	24,258	12.2	121,290	12.2
Private	12,875	6.5	64,375	6.5
Others	30,855	15.6	154,275	15.6
Region				
Large cities	63,063	31.8	315,315	31.8
Southern	82,851	41.8	414,255	41.8
Northern	47,943	24.2	239,715	24.2
Unknown	4272	2.2	21,360	2.2
Prostate cancer				
No	189,197	95.4	937,869	94.7
Yes	8932	4.6	52,776	5.3
All	198,129	100.0	990,645	100.0

The overall risk of prostate cancer in men with a family history of T2DM was 0.87 (95% CI: 0.86–0.89) as compared to matched non-exposed group (Table 2). The risk was even lower for those with more than one affected relative with T2DM, and it was 0.86 for those with two affected relatives and 0.67 for those with three and more affected relatives. Those with both parental and sibling history of T2DM has a SIR of 0.81, as compared to those with only parental history (0.95) or those with only sibling history (0.84). In addition, we examined the risk of prostate cancer among individuals with both a family history of T2DM and a personal history of T2DM, the SIR was 0.71 (*N* = 561, 95% CI: 0.65–0.77); the SIR was 0.94 (95% CI: 0.92–0.96) for those without personal history of T2DM but having at least one relative with T2DM. Sensitivity analyses in Table A1 in Appendix suggest that misclassification of exposure in the control group had limited effect on our observation.

**Table 2:** **Risk of prostate cancer among individuals with a family history of type 2 diabetes as compared to matched non-exposed group**.

Characteristics	E	O	SIR	95% CI			
Overall	10216.3	8932	**0.87**	0.86	0.89
Numbers of affected relatives					
One	8706.6	7675	**0.88**	0.86	0.90
Two	1278.3	1102	**0.86**	0.81	0.91
Three and more	231.5	155	**0.67**	0.57	0.78
Type of family history					
Parental history	3407.2	3238	**0.95**	0.92	0.98
Sibling history	5994.4	5032	**0.84**	0.82	0.86
Both parental and sibling history	814.7	662	**0.81**	0.75	0.88

*E, expected number of cases; O, observed number of cases; SIR, standardized incidence ratio, and adjusted for age, period, socioeconomic status, and region of residence; Bold type, 95% CI does not include 1.00*.

## Discussion

In this population-based nationwide cohort study, we found that the overall incidence of prostate cancer was significantly lower when first degree relatives (including parents and siblings) were diagnosed with T2DM as compared to matched controls. The incidence was even lower for those with more than one affected relatives, strongly suggesting that genetic factors that contributed to T2DM may protect against the development of prostate cancer.

One advantage of the present study is that all the data were retrieved from nationwide databases guaranteeing reliable estimation. All the prostate cancer patients were identified from a nationwide population database with high accuracy and high coverage. In addition, the study population could be followed completely. In Sweden, patients with T2DM were normally diagnosed by two doctors, one from primary health-care center, and one from specialists in the hospitals, which can guarantee high accuracy as compared to self-reported questionnaire. Many confounding factors, including age at diagnosis, socioeconomic status, and regions of residence, were adjusted in the analyses. However, a few limitations should be kept in mind when interpreting the observed associations. One limitation of this study is that we had no information about other individual-related factors, such as diet, smoking, and obesity. Another limitation is that some T2DM patients do not require hospitalization and the present results might be applicable only to hospitalized patients with probably a severe disease (20). However, low sensitivity should not lead to differential bias in the current study. In addition, a proportion of men classified as non-prostate cancer cases in the current study might have latent undiagnosed prostate cancer. However, such non-differential bias could lead to our results to null. The probability of having a family history of T2DM depends on the number of relatives, and on their respective attained ages at the time of study. Although the matching by year of birth might have attenuated the impact of family structure disparities, it might be important and necessary to include family structures in future studies.

To our knowledge, this is the first nationwide study to assess the risk of prostate cancer in relation to a family history of T2DM. The relative risk of prostate cancer was 13% lower in men with a family history of T2DM as compared to the references, which was very similar to the subsequent risk of prostate cancer in Swedish T2DM patients (adjusted SIR of 0.88) (6). Familial negative association between T2DM and prostate cancer is necessary, but not sufficient, to infer a genetic cause. Both shared genetic and environmental factors might contribute to familial aggregation. It is known that obesity is associated with T2DM, whereas obesity has also been noted to be negatively associated with non-aggressive prostate cancer and positively associated with aggressive prostate cancer (21). Families with several obese relatives will be more likely to have several members with T2DM. In such families, prostate cancer under-detection might explain a negative association with non-aggressive prostate cancer, the most common form of the disease. In addition, we found individuals with a family history of T2DM and diagnosed with T2DM had a very low risk of prostate cancer, together with the evidence that those men with more than one affected relative with T2DM had a very lower risk of prostate cancer, suggesting that genetic factors common to T2DM and prostate cancer are indeed involved, and they could contribute greatly to the current findings. Many genome-wide association studies (GWAS) were done to explore the contribution of common genetic variation on T2DM and prostate cancer (22–27), but it is still less known whether these two diseases share genetic factors. Based on our current research findings, it is highly recommended to explore the loci for both diabetes and prostate cancer that were already identified by GWAS and to understand whether there are shared genetic factors contributed for the inverse association between these two common diseases.

In summary, family history of T2DM was associated with a lower incidence of prostate cancer, and the risk was even lower for those with more than one affected relative. Our study suggests that genetic factors or shared familial factor (such as obesity) that contributed to T2DM may protect against prostate cancer, but further studies are needed to explore which genetic factors contribute to the observed familial negative association.

## Author Contributions

JJ, JS, and KS designed the study; JS and KS obtained the data; JJ did the analyses; JJ wrote the manuscript; JJ, JS, and KS approved the final manuscript. JJ was the guarantor.

## Conflict of Interest Statement

The authors declare that the research was conducted in the absence of any commercial or financial relationships that could be construed as a potential conflict of interest.

## Appendix

**Table A1:** **Adjusted SIR according to the percentage of misclassification of the exposure in the control group**.

Misclassification, %	Adjusted SIR
0.01	0.87
0.05	0.86
0.10	0.85
0.15	0.85
